# Effectiveness of Immune Checkpoint Inhibition vs Chemotherapy in Combination With Radiation Therapy Among Patients With Non–Small Cell Lung Cancer and Brain Metastasis Undergoing Neurosurgical Resection

**DOI:** 10.1001/jamanetworkopen.2022.9553

**Published:** 2022-04-29

**Authors:** David Wasilewski, Josefine Radke, Ran Xu, Matthias Raspe, Anna Trelinska-Finger, Tizian Rosenstock, Paul Poeser, Elisa Schumann, Judith Lindner, Frank Heppner, David Kaul, Norbert Suttorp, Peter Vajkoczy, Nikolaj Frost, Julia Onken

**Affiliations:** 1 Department of Neurosurgery, Charité – Universitätsmedizin Berlin, Freie Universität Berlin, Humboldt-Universität zu Berlin and Berlin Institute of Health, Berlin, Germany; 2 Department of Neuropathology, Charité – Universitätsmedizin Berlin, Freie Universität Berlin, Humboldt-Universität zu Berlin and Berlin Institute of Health, Berlin, Germany; 3 German Cancer Consortium, Heidelberg, Berlin, Germany; 4 Berlin Institute of Health at Charité – Universitätsmedizin Berlin, Berlin, Germany; 5 Department of Infectious Diseases and Pulmonary Medicine, Charité – Universitätsmedizin Berlin, Freie Universität Berlin, Humboldt-Universität zu Berlin and Berlin Institute of Health, Berlin, Germany; 6 Charité Comprehensive Cancer Center – Universitätsmedizin Berlin, Freie Universität Berlin, Humboldt-Universität zu Berlin and Berlin Institute of Health, Berlin, Germany; 7 Department of Pathology, Charité – Universitätsmedizin Berlin, Freie Universität Berlin, Humboldt-Universität zu Berlin and Berlin Institute of Health, Berlin, Germany; 8 Department of Radiation Oncology, Charité – Universitätsmedizin Berlin, Freie Universität Berlin, Humboldt-Universität zu Berlin and Berlin Institute of Health, Berlin, Germany

## Abstract

**Importance:**

Patients with brain metastases from non–small cell lung cancer (NSCLC) have regularly been excluded from prospective clinical trials that include therapy with immune checkpoint inhibitors (ICIs). Clinical data demonstrating benefit with ICIs, specifically following neurosurgical brain metastasis resection, are scarce.

**Objective:**

To evaluate and compare the association of radiation therapy with ICIs vs classic therapy involving radiation therapy and chemotherapy regarding overall survival in a cohort of patients who underwent NSCLC brain metastasis resection.

**Design, Setting and Participants:**

This single-center 1:1 propensity-matched comparative effectiveness study at the largest neurosurgical clinic in Germany included individuals who had undergone craniotomy with brain metastasis resection from January 2010 to December 2021 with histologically confirmed NSCLC. Of 1690 patients with lung cancer and brain metastasis, 480 were included in the study. Key exclusion criteria were small-cell lung cancer, lack of tumor cells by means of histopathological analysis on brain metastasis resection, and patients who underwent biopsy without tumor resection. The association of overall survival with treatment with radiation therapy and chemotherapy vs radiation therapy and ICI was evaluated.

**Exposures:**

Radiation therapy and chemotherapy vs radiation therapy and ICI following craniotomy and microsurgical brain metastasis resection.

**Main Outcomes and Measures:**

Median overall survival.

**Results:**

From the whole cohort of patients with NSCLC (N = 384), 215 (56%) were male and 169 (44%) were female. The median (IQR) age was 64 (57-72) years. The 2 cohorts of interest included 108 patients (31%) with radiation therapy and chemotherapy and 63 patients (16%) with radiation therapy and ICI following neurosurgical metastasis removal (before matching). Median (IQR) follow-up time for the total cohort was 47.9 (28.2-70.1) months with 89 patients (23%) being censored and 295 (77%) dead at the end of follow-up in December 2021. After covariate equalization using propensity score matching (62 patients per group), patients receiving radiation therapy and chemotherapy after neurosurgery had significantly lower overall survival (11.8 months; 95% CI; 9.1-15.2) compared with patients with radiation therapy and ICIs (23.0 months; 95% CI; 20.3-53.8) (*P* < .001).

**Conclusions and Relevance:**

Patients with NSCLC brain metastases undergoing neurosurgical resection had longer overall survival when treated with radiation therapy and ICIs following neurosurgery compared with those receiving platinum-based chemotherapy and radiation. Radiation and systemic immunotherapy should be regularly evaluated as a treatment option for these patients.

## Key Points

**Question:**

What is the effect of radiation combined with immunotherapy in terms of survival in patients with non–small cell lung cancer (NSCLC) brain metastases after neurosurgical brain metastasis resection?

**Findings:**

This comparative effectiveness study of 171 patients from a total cohort of 384 individuals with NSCLC brain metastases who had undergone neurosurgical brain metastasis resection found an association between improved median overall survival and subsequent radiation and immunotherapy compared with patients receiving radiation and platinum-based chemotherapy.

**Meaning:**

These findings suggest that patients with resected NSCLC brain metastases may benefit from subsequent treatment with immune checkpoint inhibitors; regular and rigorous interdisciplinary evaluation of these patients for combined radiation and immunotherapy following neurosurgical care is advised.

## Teaser

This study evaluates the overall survival in patients with non–small cell lung cancer brain metastases receiving radiation therapy with immune checkpoint inhibition following resection surgery vs those receiving radiation therapy and chemotherapy following resection surgery.

## Introduction

Lung cancer represents one of the leading causes of death with non–small cell lung cancer (NSCLC), accounting for approximately 85% of patient cases.^1,2^ Brain metastases are frequent, with up to 50% of patients developing brain metastasis within the course of their disease.^2,3^ Additionally, at least 10% of patients present with brain metastases at initial clinical presentation.^4^ Historically, brain metastases have been regarded as a terminal disease stage harboring a poor prognosis with a median overall survival (OS) of 3 months with best supportive care or up to 6 months with whole brain radiation therapy and up to 8 months in selected patients undergoing surgical metastasis removal combined with adjuvant treatment.^1,4,5^ In a subset of patients with good functional status and surgically accessible or symptomatic brain metastases, aggressive treatment—including craniotomy with neurosurgical resection followed by local irradiation and systemic treatment—is common practice.^4,5,6^ However, there is a lack of specific and prospective randomized studies in the context of neurosurgically treated patients comparing different treatment options, including local therapy and systemic treatment using chemotherapy or immune checkpoint inhibitors (ICIs). More evidence is available for upfront local radiation and systemic treatment with either ICIs or small molecule inhibitors.^7,8,9,10,11^ Retrospective data may help in further evaluating the role of local and systemic treatment modalities specifically in cohorts of surgically treated patients.^11,12^ This retrospective single-center comparative effectiveness study describes clinical, radiological, and histological characteristics; prognostic factors; and OS in a large cohort of patients with NSCLC brain metastases who underwent craniotomy and brain metastasis resection. We compare outcomes in patients treated with radiation therapy and chemotherapy vs those receiving radiation therapy and ICI after surgical brain metastasis removal using a 1:1 propensity score matching approach to account for bias and heterogeneity among the patient groups.^13^

## Methods

### Patient Cohort and Study Variables

**Figure 1.**
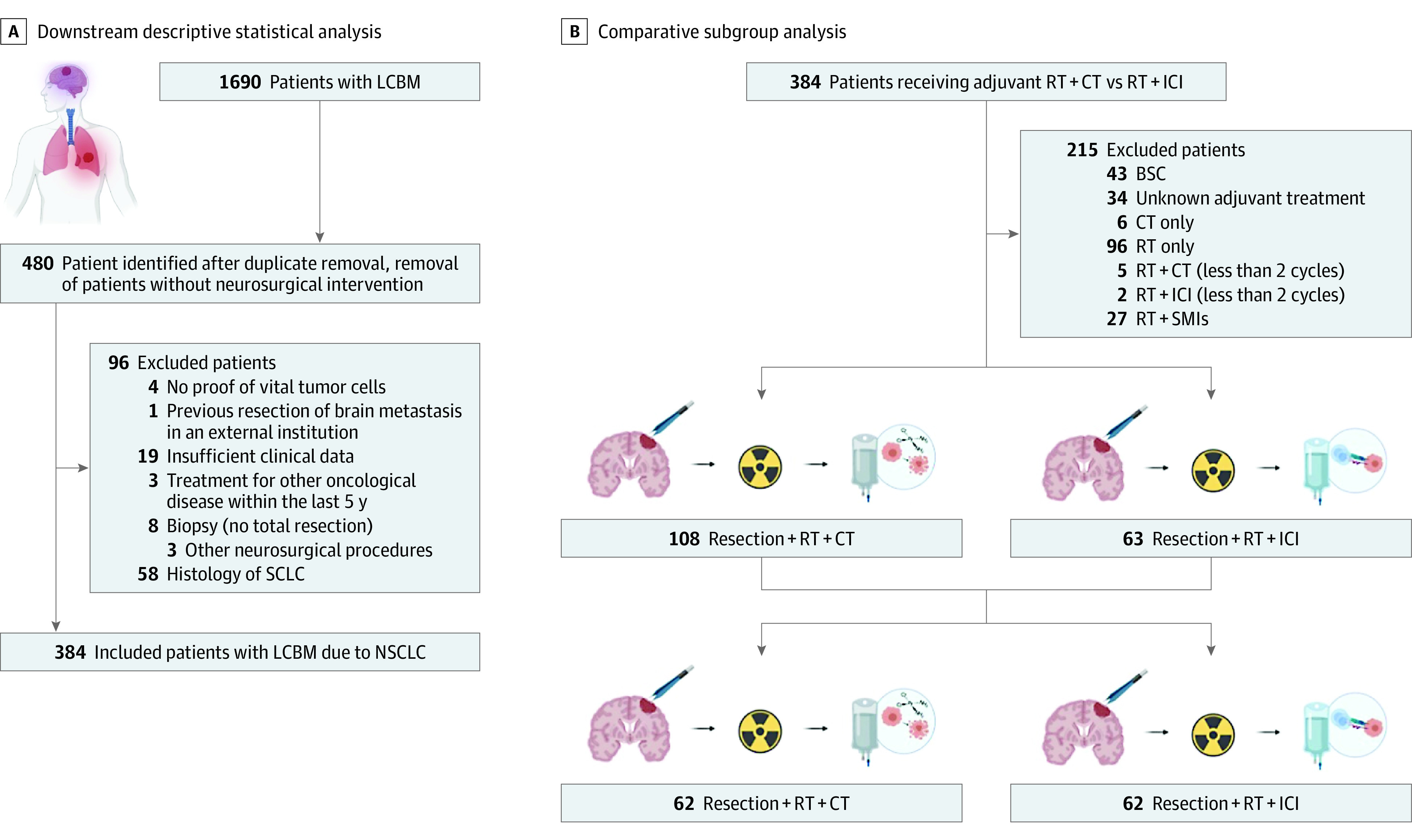
Creation of Cohorts of Interest and Design of the Study BSC indicates best supportive care; CT, chemotherapy; ICI, immune checkpoint inhibitors; LCBM, lung cancer brain metastases; NSCLC, non–small cell lung cancer; RT, radiation therapy; SCLC, small cell lung cancer; SMI, small-molecule inhibitors.

This single-center retrospective study involved all 3 hospital sites of the Charité University Medical Hospital from January 2010 to September 2021, with data censoring December 31, 2021. Patient data were identified using an institutional database (SAP, Walldorf, Germany) as well as the Charité Comprehensive Cancer Center Registry. Patients with a histopathological confirmation of NSCLC from both an intracerebral and primary tumor site from January 2010 and December 2021 were included. The presented study was conducted in accordance with the ethical standards outlined in the Declaration of Helsinki. We also obtained a positive vote by the local committee beforehand (EA1/399/20). To guide further treatment options, patients’ circumstances were regularly discussed in an interdisciplinary tumor board after surgical brain metastasis resection at a point when histopathological analysis of resected diseased central nervous system tissue was completed. Of 480 patients with neurosurgical intervention, 96 were excluded from further analysis. Exclusion criteria included no proof of vital tumor cells on tissue analysis (eg, inflammatory or radiogenic changes and hemorrhage without signs of vital tumor cells) (n = 4); if patients underwent stereotactic biopsy, navigated biopsy, or subtotal resection only (n = 7); ventriculocisternostomy or ventriculoperitoneal-shunt implantation (n = 3); presence of SCLC (n = 58); and previous brain surgery in an external institution (n = 1). Exclusion was necessary in case of insufficient clinical information (n = 19) or in case patients received treatment because of any other malignant neoplasm prior to their diagnosis of lung cancer brain metastasis (n = 3) (Figure 1). The primary outcome was median OS, defined as time from brain metastasis resection until death from any cause. Baseline was defined as time of first brain metastasis resection; baseline characteristics were selected according to previous retrospective studies.^2,5,8,11^ Karnofsky performance status and NSCLC-specific gradual prognostic assessment index using molecular markers were assessed after first brain metastasis resection (baseline).^12^ Radiological baseline variables included anatomical location of brain metastases, number of brain metastases, volume, presence of hemorrhage, leptomeningeal disease, hydrocephalus, or presence of extracranial metastases at baseline. Radiographic images and correspondent reports from board-certified radiologists, including cranial magnetic resonance imaging, were reviewed. Magnetic resonance imaging scans were subjected to further image analysis in case no written reports were available. Presence of extracranial disease was assessed via computerized tomography scans of the chest, abdomen, and pelvis or whenever available via whole-body positron emission tomography scans. Treatment-related baseline parameters included total number of brain metastasis resections, resection of primary tumor mass, status of pretreatment at baseline, and adjuvant therapy after brain metastasis resection. Information on systemic treatment and radiotherapy before or after first brain metastasis resection at our institution were retrieved from our database and patient records. All patients of both groups (radiation therapy with chemotherapy and radiation therapy with ICIs) completed treatment following surgery, including radiation (either local radiation, whole brain radiation, or stereotactic radiosurgery) and at least 2 cycles of either platinum-based chemotherapy or immunotherapy (Tables 1 and 2). Follow-up data were obtained until December 2021. Biomarkers and histopathological characteristics included lymphocyte and neutrophil count ranging from 3 weeks before to 3 weeks after first brain metastasis resection, driver mutational status, extracranial and intracranial Ki67, programmed cell death ligand 1, and tumor proportion score were assessed. Institutional pathological review was mandatory, and all resected specimens were reviewed by board-certified neuropathologists and pathologists for diagnosis. Informed consent was waived owing to the retrospective nature of the study.

**Table 1.** Comparison Before Propensity Score Matching: Patients Without vs With Immune Therapy With Checkpoint-inhibitors After Removal of Brain Metastases

Characteristic	Overall (N = 171)	Patients by treatment group, No. (%)		*P* value^a^
		Radiation therapy + chemotherapy (n = 108)	Radiation therapy + immune checkpoint inhibitors (n = 63)	
Sex				.14
Female	86 (50)	59 (55)	27 (43)	
Male	85 (50)	49 (45)	36 (57)	
Age, median (IQR), y	62 (56-70)	61 (55-68)	63 (58-71)	.14
Brain metastases, No.				
1	125 (73)	75 (69)	50 (79)	.20
>1	46 (27)	33 (31)	13 (21)	
Brain metastases treatments, No.				
1	151 (88)	97 (90)	54 (86)	.40
2	20 (12)	11 (10)	9 (14)	
Location of brain metastases				
Supratentorial	106 (62)	66 (61)	40 (63)	.93
Infratentorial	27 (16)	17 (16)	10 (16)	
Both	38 (22)	25 (23)	13 (21)	
Primary tumor resection				
No	129 (75)	78 (72)	51 (81)	.20
Yes	42 (25)	30 (28)	12 (19)	
Pretreatment				
No	139 (81)	87 (81)	52 (83)	.70
Yes	32 (19)	21 (19)	11 (17)	
Adjuvant radiation therapy				
Stereotactic radiosurgery	31 (18)	20 (19)	11 (17)	.90
Other than stereotactic radiosurgery	140 (82)	88 (81)	52 (83)	
Radiation therapy dose, Gy				
<30	62 (36)	38 (36)	24 (38)	.70
≥30	108 (64)	69 (64)	39 (62)	
Unknown^b^	1	1	0	
GPA score				
Bad	98 (57)	67 (62)	31 (49)	.10
Good	73 (43)	41 (38)	32 (51)	
UICC stage				
IV	17 (10)	11 (10)	6 (9.7)	.93
Other than IV	152 (90)	96 (90)	56 (90)	
Unknown^b^	2	1	1	
Extracranial metastasis	62 (36)	37 (34)	25 (40)	.50
Volume, mL				
<15	64 (38)	34 (32)	30 (48)	.04
≥15	106 (62)	73 (68)	33 (52)	
Unknown^b^	1	1	0	
PD-L1 intracranial				
<1%	49 (58)	27 (79)	22 (43)	<.001
≥1%	36 (42)	7 (21)	29 (57)	
Unknown^b^	86	74	12	
Ki-67 intracranial				
<1%	84 (57)	43 (50)	41 (66)	.05
≥1%	64 (43)	43 (50)	21 (34)	
Unknown^b^	23	22	1	
PD-L1 extracranial				
<1%	19 (35)	8 (36)	11 (33)	.80
≥1%	36 (65)	14 (64)	22 (67)	
Unknown^b^	116	86	30	
Ki-67 extracranial				
<1%	22 (44)	8 (38)	14 (48)	.50
≥1%	28 (56)	13 (62)	15 (52)	
Unknown^b^	121	87	34	
NLR				
<5	62 (42)	36 (40)	26 (45)	.50
≥5	87 (58)	55 (60)	32 (55)	
Unknown^b^	22	17	5	
TTF1 status				
Negative	56 (34)	39 (37)	17 (28)	.20
Positive	111 (66)	67 (63)	44 (72)	
Unknown^b^	4	2	2	

Abbreviations: GPA, gradual prognostic assessment; NLR, neutrophil-to-lymphocyte ratio; PD-L1, programmed cell death ligand 1; TTF1, thyroid transcription factor 1; UICC, Union for International Cancer Control.

^a^ χ^2^ test of independence was used to analyze the frequency for categorical variables. Nonparametric Wilcoxon rank sum test was used for comparing 2 means not normally distributed; when expected count was below 5, Fisher exact test was used.

^b^ Unknown data are not factored into the percentage distribution of the other rows with respect to a given covariate in order to discriminate better between different groups.

**Table 2.** Summary of Balance for Matched Data With Patients Either Receiving Subsequent Radiation and Chemotherapy or Radiation and Immune Therapy After Performing Propensity Score Matching

Characteristic	Overall (N = 124)	Patients by treatment group, No. (%)		SMD	*P* value^a^
		Radiation therapy + chemotherapy (n = 62)	Radiation therapy + immune checkpoint inhibitors (n = 62)		
Sex					
Female	62 (50)	36 (58)	26 (42)	0.098	.07
Male	62 (50)	26 (42)	36 (58)		
Age, median (IQR), y	62 (57-71)	62 (57-72)	63 (57-70)	0.075	.60
Brain metastases, No.					
1	101 (81)	52 (84)	49 (79)	−0.12	.50
>1	23 (19)	10 (16)	13 (21)	0.12	
Volume, mL					
<15	56 (45)	27 (44)	29 (47)	0.06	.70
≥15	68 (55)	35 (56)	33 (53)	−0.06	
Location of brain metastases					
Supratentorial	82 (66)	43 (69)	39 (63)	−0.13	.70
Infratentorial	19 (15)	9 (15)	10 (16)	0.04	
Both	23 (19)	10 (16)	13 (21)	0.12	
GPA score					
Bad	61 (49)	31 (50)	30 (48)	−0.03	.90
Good	63 (51)	31 (50)	32 (52)	0.03	
Extracranial metastasis	51 (41)	26 (42)	25 (40)	−0.03	.90
Adjuvant radiation therapy					
Stereotactic radiosurgery	24 (19)	13 (21)	11 (18)	−0.08	.60
Other than stereotactic radiosurgery	100 (81)	49 (79)	51 (82)	0.08	
Primary tumor resection					
No	101 (81)	50 (81)	51 (82)	0.04	.80
Yes	23 (19)	12 (19)	11 (18)	−0.04	
Radiation dose, Gy					
<30	44 (35)	21 (34)	23 (37)	0.07	.80
≥30	80 (65)	41 (66)	39 (63)	−0.07	
UCC stage					
Other than IV	115 (93)	59 (95)	56 (90)	−0.16	.50
IV	9 (7.3)	3 (4.8)	6 (9.7)	0.16	

Abbreviations: GPA, gradual prognostic assessment; SMD, standarized mean difference; UICC, International Cancer Control.

^a^ χ^2^ test of independence was used to analyze the frequency for categorical variables. Nonparametric Wilcoxon rank sum test was used for comparing 2 means not normally distributed; when expected count was below 5, Fisher exact test was used.

### Statistical Analysis

We used R version 1.1.442 (R Foundation) to compute descriptive statistics, including frequencies, means, and SDs, to characterize the cohort. Baseline characteristics or continuous data were compared across cohorts using Wilcoxon rank-sum tests, while categorical data were compared using Fisher exact or χ^2^ tests. The gtsummary package (R Foundation) was used to describe tabular data of the patient cohort, including categorical and numerical variables. Median OS was estimated by Kaplan-Meier analysis with 95% CI bands being displayed in gray; plotting was performed using the survival and survminer packages (R Foundation). The prognostic value of each variable was tested using log-rank. Univariable and multivariable Cox regression modeling served to assess the effect of 1 or multiple clinical variables on OS and was done using the survival and survminer packages (R Foundation). To estimate the effect of treatment and balance covariate distribution, propensity score matching was performed based on a generalized linear model that implements logistic regression by the nearest-neighbor matching method with a 1:1 matching ratio and a caliper set to 0.05.^13^ Propensity score matching involved the following baseline covariates: sex, age, number of brain metastases at baseline, volume and location of brain metastases, gradual prognostic assessment score, status of extracranial disease burden, mode of adjuvant radiation therapy, status of primary tumor resection, dose of adjuvant radiation therapy, and baseline Union for International Cancer Control (UICC) stage (eTable 1 in the Supplement). Before propensity score matching, these covariates were assessed in terms of their association with OS. Further R packages included dplyr, tidyverse, swimplot, MatchIt, and WeightIt.^13^ Data collection was done with Excel version 14.3.9 (Microsoft). A *P* value <.05 was considered significant with *P* values being 2-sided. R code and raw data will be made available on request.

## Results

### Baseline Characteristics

Characteristics of patients with lung cancer brain metastases who met the inclusion criteria (N = 384) are shown in eTable 1 in the Supplement. There were 215 (56%) male and 169 (44%) female individuals in the total cohort of patients. The median (IQR) age was 64 (57-72) years. Eighty-seven patients (23%) underwent upfront resection of their primary lung tumor, whereas in 35 patients (9%), primary tumor resection occurred after first brain metastasis resection (eTable 1 in the Supplement). Forty patients (11%) underwent more than 1 brain metastasis resection: 28 of these patients (70%) underwent reoperation because of local disease recurrence and 12 (30%) because of independent brain metastases. After first brain metastasis resection, 43 patients (11%) received best supportive care, 6 (2%) received chemotherapy only, 103 patients (27%) adjuvant radiation therapy only, and 108 patients (28%) adjuvant radiation therapy and chemotherapy. Finally, 63 patients (16%) were treated with radiation therapy and ICIs, and 27 patients (7%) received radiation therapy with small molecule inhibitors. In 34 patients (9%), there was a lack of information on adjuvant treatment (eTable 1 in the Supplement). Median (IQR) follow-up time was 47.9 (28.2-70.1) months with 89 patients (23%) being censored and 295 (77%) being dead at the end of follow-up in December 2021. Cumulated median OS of the whole cohort was 10.1 months (95% CI, 8.67-11.8) (eFigure 1 in the Supplement). Other clinical, radiological, biomarker-related, and histopathological characteristics of this collective are summarized in eTable 1 in the Supplement. Before propensity score matching, statistical testing was performed using Spearman rank correlation (eFigure 2 in the Supplement) and univariable and multivariable Cox proportional hazard (eTable 2 in the Supplement) regression for covariates of potential prognostic relevance. Correlation between scaled Schoenfeld residuals and time were used to test the proportional hazards assumption (eFigure 3 in the Supplement). Baseline characteristics, including sex, age, number of brain metastases, volume and location of brain metastases, gradual prognostic assessment score, status of extracranial disease burden, mode of adjuvant radiation therapy, status of primary tumor resection, dose of adjuvant radiation therapy, and baseline UICC stage, were chosen as covariates relevant for later propensity score matching. Primary tumor resection (hazard ratio [HR], 0.51; 95% CI, 0.32-0.82; *P* = .006) and presence of extracranial metastases (HR, 2.17; 95% CI, 1.34-3.51; *P* = .01), and radiation therapy with ICI following brain metastasis resection (HR, 0.32; 95% CI, 0.20-0.51; *P* < .001) were independently associated with OS (eTable 2 in the Supplement).

### Description of the Unmatched Patient Cohort

**Figure 2.**
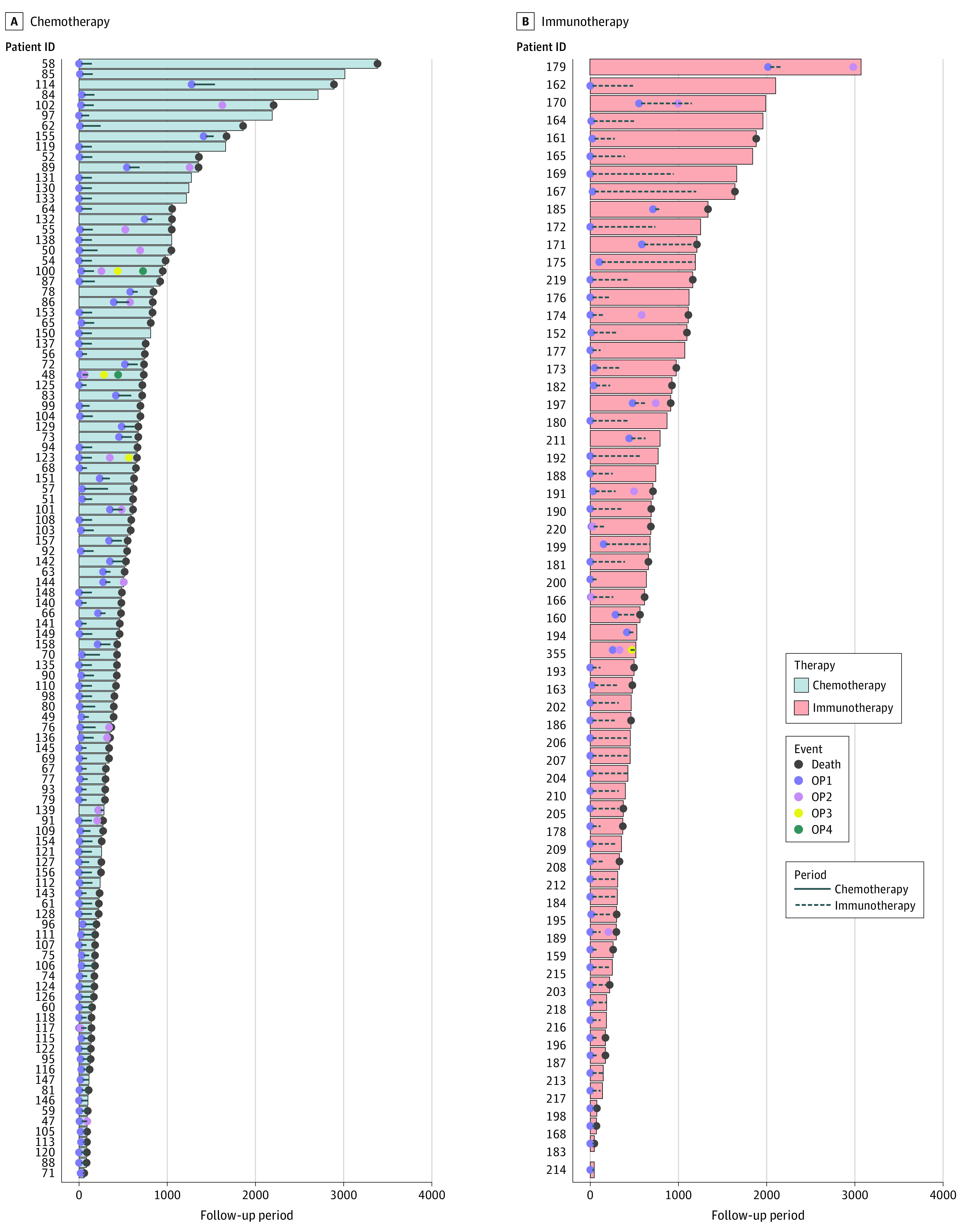
Swimmer Plot Representation of Overall Survival of Matched Patients ID indicates identification; OP, operation.

**Figure 3.**
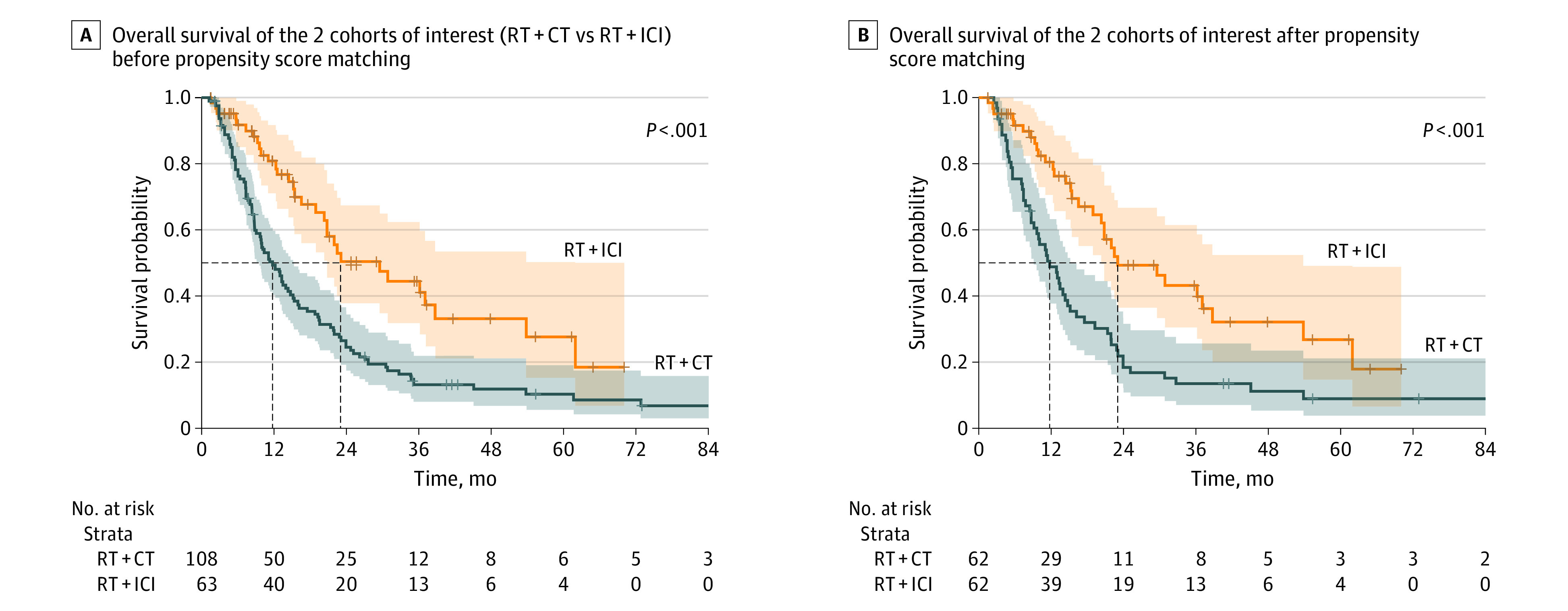
Kaplan-Meier Curves for Overall Survival Among Matched and Unmatched Data CT indicates chemotherapy; ICI, immune checkpoint inhibitors; RT, radiation therapy.

Unmatched groups were plotted using a swimmer plot (Figure 2) and did not differ significantly in terms of prognostic covariates (eg, of primary tumor resection, baseline UICC status, number of brain metastases at baseline, or presence of extracranial metastases) (Table 1). In the unmatched setting, patients receiving radiation therapy and chemotherapy after neurosurgery had a median OS of 10.4 months (95% CI, 7.4-14.7) compared with 23.0 months in patients receiving radiotherapy and ICIs (95% CI, 20.3-53.8; *P* < .001) (Figure 3A). In patients in the chemotherapy group, 53 received a combination of carboplatin plus another agent (etoposide, gemcitabine, pemetrexed, or taxol), and 52 received cisplatin plus (etoposide, gemcitabine, or pemetrexed); the remaining 3 patients received single-agent chemotherapy only. In the group of patients receiving ICIs, 37 (59%) received pembrolizumab, 7 (11%) received atezolizumab, and 6 (10%) nivolumab; the remaining portion of patients received carboplatin, pemetrexed, and pembrolizumab as a combined therapy (data not shown).

### Description of the Matched Patient Cohort and Prognostic Factors

After matching (62 patients per group), patients receiving adjuvant radiation therapy and chemotherapy had a significantly decreased median OS (11.8 months; 95% CI, 9.1-5.2) compared with patients receiving radiation therapy and ICIs (23.0 months; 95% CI, 20.3-3.8 months; *P* < .001) following brain metastasis resection (Figure 3B). Accordingly, the matching process for covariate balancing eliminated significant differences between both groups, leaving no covariate differing significantly between compared groups as illustrated by the distribution of the propensity scores for both groups and mean and SD values for each of prognostically relevant covariates (Table 2; eFigure 5 in the Supplement). Additionally, Kaplan-Meier survival analysis for the matched patient data showed that presence of extracranial metastases, gradual prognostic assessment score, primary tumor resection status, number of brain metastases at baseline were all significantly associated with OS (eFigure 4 in the Supplement). After propensity score matching, we performed univariable and multivariable analysis for the matched data set to associate covariates of interest with OS in both cohorts. Multivariable Cox regression for baseline covariates of matched patients in this cohort identified primary tumor resection (HR, 0.39; 95% CI, 0.20-0.74; *P* = .004) and presence of extracranial metastases (HR, 1.92; 95% CI, 1.16-3.17; *P* = .01) as independent prognostic factors. In addition, radiation therapy and ICI following brain metastasis resection was also an independent prognostic factor (HR, 0.34; 95% CI, 0.21-0.55; *P* < .001), whereas other clinical variables were not associated with OS by means of multivariable analysis (eTable 3 in the Supplement).

## Discussion

To our knowledge, this is the largest retrospective single-center analysis providing survival data on patients with NSCLC receiving radiation therapy and ICI therapy following brain metastasis resection. Here, 1:1 nearest-neighbor propensity score matching showed a significant association with OS in patients receiving adjuvant radiation therapy and ICIs compared with patients receiving radiation therapy and chemotherapy following surgery (median OS, 23.0 months vs 11.8 months, respectively) (Figure 3). This effect was corroborated by means of multivariable Cox regression analysis where treatment with radiation therapy and ICIs following lung cancer brain metastasis resection was significantly associated with a decreased hazard for death (eTable 2 in the Supplement). Previous studies evaluating the effect of novel ICI treatment regimens following brain metastasis resection were largely focused on melanoma and did not specifically address the patient subgroup undergoing brain metastasis resection.^5,14,15,16^ For example, Bander et al^5^ reported on a large melanoma brain metastasis cohort in which, independent of brain metastasis resection, patients treated with novel immune or targeted therapies experienced longer OS compared with patients with melanoma brain metastasis diagnosed in the preimmune or pretargeted therapy era (13 vs 7 months; *P* < .001). Additionally, recent retrospective data^17^ from patients with melanoma brain metastasis showed that patients who were immunotherapy-naive undergoing upfront surgical brain metastasis resection followed by immunotherapy had higher survival compared with patients who were pretreated with immune therapy before neurosurgical resection. Patients with NSCLC brain metastases who experienced primary tumor resection showed an increase in OS compared with patients who had not undergone resection on their primary tumor (eTables 1 and 2 and eFigure 4 in the Supplement), which is in line with previous observations of the impact of local ablative therapy in patients with oligometastatic disease.^18,19,20,21^ This in turn indicates the potential benefit of upfront primary tumor resection in eligible patients.^18,19,20,21^ The possible benefit of surgery with adjuvant radiation therapy and ICIs is likely based on biologic effects mediated by radiation-induced cancer cell damage with subsequent release of tumor antigens and blockade of immunosuppressive signaling.^7,9,16,20,22^ Removal of a relevant tumor mass by means of microsurgical brain metastasis resection likely augments the impact of the additive effect (abscopal effect) of radiation therapy and ICIs in our study and might contribute to epitope spreading.^10,11,15,18,19,20,21,22,23,24,25^ Besides limited evidence on treatment modalities following brain metastasis resection, there are insufficient data on the direct comparison of resection and radiation therapy vs radiation therapy alone. Yet, a prospective randomized clinical study^26^ found that radiation therapy with stereotactic radiosurgery was associated with a decreased 12-month recurrence rate compared with surgery alone.^19,21,27^ Although our study included only patients who had undergone surgery with a relatively large lesion volume not amenable to radiation, it would be interesting to assess and compare the effect of surgery with radiation therapy and ICIs vs radiation therapy and ICIs in a prospective setting. However, the observed benefit with systemic ICIs together with radiation therapy in patients undergoing surgical lung cancer brain metastasis resection seems to be in accordance with previously published data on melanoma brain metastasis. More comprehensive analyses are needed to compare the different adjuvant combinatory regimens with ICIs following brain metastasis removal not only in melanoma and lung cancer, but also in other cancer types that frequently give rise to brain metastasis, such as renal cell cancer or breast cancer.^19,20,21,22,25^ Prospective randomized clinical trials on surgical patients exploiting ICIs combined with chemotherapy and radiation therapy should evaluate OS as well as intracranial response according to the immunotherapy Response Assessment in Neuro-Oncology (iRANO) criteria or brain metastasis (RANO-BM) criteria. Additionally, the systemic response is crucial in these patients and should be evaluated by the iRANO criteria.^28^ Immune monitoring or evaluation of experimental (biomarker) end points by deploying multiplex approaches before or during treatment might provide insights into the dynamics of immune cells in these patients and potentially lead to discovery of new biomarkers.^22,25,29,30,31^

### Limitations

This study has limitations. Given the retrospective nature of the current study, there are several limitations. Although improvement of OS with surgery following radiation therapy and ICIs is likely associated with superior intracranial and extracranial disease control, we do not provide data with respect to cause of death, intracranial and extracranial progression-free survival, and extent of brain metastasis resection because of incomplete follow-up documentation. Further, data on important biomarkers, including programmed cell death ligand 1 status, epidermal growth factor receptor, and anaplastic lymphoma kinase, are incomplete, which can be explained by the long observation period (2010-2021) and shows an inherent limitation of clinical data. Although propensity score matching is an adequate statistical solution for balancing covariates of treatment and control groups, the relatively small sample size with only 63 patients in the ICI group is also a limiting factor of the study.

## Conclusions

With rising incidence of lung cancer brain metastases, the number of patients needing aggressive local ablative therapy (ie, potential neurosurgical candidates) combined with systemic therapy will therefore likely rise as well.^20,21,22^ Although patients undergoing surgical brain metastasis removal are regularly treated with different systemic therapies, prospective randomized clinical studies are lacking for this patient cohort. In this comparative effectiveness study using propensity score matching on a relatively large data set of patients with NSCLC who underwent brain metastasis treatment following surgery, radiation and use of ICIs was associated with greater OS compared with classic platinum-based chemotherapy and radiation. Our results suggest a potential benefit with ICI use in this patient cohort and highlight the importance of combinatory and interdisciplinary treatment approaches in patients with brain metastasis.

## Notes

Supplement. **eTable 1.** Demographic, clinical and radiologic characteristics at baseline **eFigure 1.** Kaplan Meier survival estimates of the whole patient cohort **eFigure 2.** Correlation matrices of radiological and biomarker-related characteristics on survival **eTable 2.** Univariable and multivariable Cox proportional regression in the whole patient cohort **eFigure 3.** Testing for the proportional hazards (PH) assumption with Schoenfeld residuals **eFigure 4.** Stratified Kaplan-Meier curves in matched patients for overall survival **eFigure 5.** Distribution of propensity scores and standardized mean differences **eTable 3.** Univariable and multivariable Cox proportional regression in the matched cohort
