# Supplementary material for: Effectiveness of Immune Checkpoint Inhibition vs Chemotherapy in Combination With Radiation Therapy Among Patients With Non–Small Cell Lung Cancer and Brain Metastasis Undergoing Neurosurgical Resection
Source: JAMA Netw Open. 2022 Apr 29;5(4):e229553. doi: 10.1001/jamanetworkopen.2022.9553 (PMC9055459; doi:10.1001/jamanetworkopen.2022.9553)
Supplement: Supplement. — eTable 1. Demographic, clinical and radiologic characteristics at baseline eFigure 1. Kaplan Meier survival estimates of the whole patient cohort eFigure 2. Correlation matrices of radiological and biomarker-related characteristics on survival eTable 2. Univariable and multivariable Cox proportional regression in the whole patient cohort eFigure 3. Testing for the proportional hazards (PH) assumption with Schoenfeld residuals eFigure 4. Stratified Kaplan-Meier curves in matched patients for overall survival eFigure 5. Distribution of propensity scores and standardized mean differences eTable 3. Univariable and multivariable Cox proportional regression in the matched cohort [file jamanetwopen-e229553-s001.pdf]

Supplemental Online Content

Wasilewski D, Radke J, Xu R, et al. Effectiveness of immune checkpoint inhibition vs chemotherapy in combination with radiation therapy among patients with non–small cell lung cancer and brain metastasis undergoing neurosurgical resection. *JAMA Netw Open*. 2022;5(4):e229553. doi:10.1001/jamanetworkopen.2022.9553

eTable 1. Demographic, clinical and radiologic characteristics at baseline

eFigure 1. Kaplan Meier survival estimates of the whole patient cohort

eFigure 2. Correlation matrices of radiological and biomarker-related characteristics on survival

eTable 2. Univariable and multivariable Cox proportional regression in the whole patient cohort

eFigure 3. Testing for the proportional hazards (PH) assumption with Schoenfeld residuals

eFigure 4. Stratified Kaplan-Meier curves in matched patients for overall survival

eFigure 5. Distribution of propensity scores and standardized mean differences

eTable 3. Univariable and multivariable Cox proportional regression in the matched cohort

This supplemental material has been provided by the authors to give readers additional information about their work.

© 2022 Wasilewski D et al. *JAMA Network Open.*

eTable 1. Demographic, clinical and radiologic characteristics at baseline

Table 1: Patient characteristics	
**Characteristic**	**N = 384**
**Gender, n (%)**	
M	215 (56)
W	169 (44)
**Age, Median (IQR)**	64 (57 – 72)
**anatomical localization, n (%)**	
frontal	121 (32)
parietal	75 (20)
temporal	40 (10)
occipital	42 (11)
cerebellar	99 (26)
other	6 (1.6)
Unknown	1
**hydrocephalus at baseline, n (%)**	47 (12)
Unknown	8
**hemorrhage at baseline, n (%)**	71 (19)
Unknown	8
**LMM at baseline, n (%)**	8 (2.1)
Unknown	8

© 2022 Wasilewski et al. *JAMA Network Open.*

Table 1: Patient characteristics	
**Characteristic**	**N = 384**
**no_of_BrM_Rxs, n (%)**	
1	344 (90)
2	34 (8.9)
3	3 (0.8)
4	3 (0.8)
**Number of brain metastases at baseline, n (%)**	
one	144 (38)
two	127 (33)
more than two	113 (29)
**Brain region affected of brain metastasis, n (%)**	
supratentorial	234 (61)
infratentorial	60 (16)
both	90 (23)
**volume of dominant (resected) brain metastasis (mL), Median (IQR)**	8 (4 – 16)
Unknown	12
**Extracranial metastasis at baseline, n (%)**	
UICC stage IV	318 (86)
UICC stage I-III	52 (14)
Unknown	14
**UICC stage, n (%)**	52 (14)

© 2022 Wasilewski et al. *JAMA Network Open.*

Table 1: Patient characteristics	
**Characteristic**	**N = 384**
Unknown	14
**KPS after operation, n (%)**	
< 70%	160 (42)
>/= 70%	224 (58)
**GPA after operation , n (%)**	
>/=2	231 (60)
<2	151 (40)
Unknown	2

eTable 1A: Demographic, clinical and radiologic characteristics at baseline

Baseline characteristics were selected based on previous reports and literature within the field[1-4]. The *gtsummary* package and windows word were used to describe tabular data of our patient cohort, including continuous, categorical or numerical as well as dichotomized variables.

Table 2: Treatment-related patient characteristics	
**Characteristic**	**N = 384**
**Number of brain metastasis resections, n (%)**	
1	344 (90)
2	34 (8.9)
3	3 (0.8)
4	3 (0.8)

© 2022 Wasilewski et al. *JAMA Network Open.*

**resection of independent lesions, n (%)**	12 (30)
**resection due to local recurrence, n (%)**	28 (70)
**time to local re-resection, Median (IQR)**	234 (72 – 503)
**primary tumor resection in context of brain metastasis resection, n (%)**	
no primary tumor resection	262 (68)
primary tumor resection before brain metastasis resection	87 (23)
primary tumor resection after brain metastasis resection	35 (9.1)
**primary tumor resection, n (%)**	
no primary tumor resection	262 (68)
primary tumor resection	122 (32)
**pre-treatment at baseline, n (%)**	
no neoadjuvant treatment	270 (70)
RTx + CTx	94 (24)
RTx + ICI	12 (3.1)
RTx + SMI	8 (2.1)
**mode of cranial radiation after first brain metastasis resection, n (%)**	
WBRT	120 (36)
local irradiation of resection cavity	88 (26)
SRS	0 (0)
not known	129 (38)

© 2022 Wasilewski et al. *JAMA Network Open.*

**fraction of stereotactically irradiated patients, n (%)**	
other than SRS	340 (89)
SRS	44 (11)
**dose of cranial radiation after first brain metastasis resection, Median (IQR)**	31 (30 – 42)
**adjuvant treatment groups of interest: RTx + CTx vs. RTx + ICI, n (%)**	
RTx + CTx	108 (63)
RTx + ICI	63 (37)
**main adjuvant treatment groups, n (%)**	
RTx + CTx	108 (57)
RTx + ICI	63 (33)
RTx + SMIs	19 (10)
**adjuvant treatment modalities, n (%)**	
best supportive care (bsc)	43 (11)
chemotherapy (CTx)	6 (1.6)
not known	34 (8.9)
radiation (RTx)	103 (27)
radiation and chemotherapy(RTx + CTx)	108 (28)
radiation and immune therapy (RTx + ICI)	63 (16)
radiation and targeted therapy (RTx + SMI)	27 (7.0)

eTable 1B: Treatment-related patient characteristics at baseline.

© 2022 Wasilewski et al. *JAMA Network Open.*

**Table 3: Histopathological and biomarker-related patient characteristics**	
**Characteristic**	**N = 384**
**neutrophil-to-lymphocyte ratio, n (%)**	
< 5	105 (35)
>/= 5	195 (65)
Unknown	84
**tumor subtype, n (%)**	
NSCLC	384 (100)
**histologic subtype, n (%)**	
adenocarcinoma	329 (86)
adenosquamous	4 (1.0)
neuroendocrine differentiation	13 (3.4)
pulmonary sarcomatoid carcinoma	2 (0.5)
squamous cell cancer	36 (9.4)
**PDL1 status brain metastasis tissue, n (%)**	
< 1	90 (56)
>/= 1	72 (44)
Unknown	222
**Ki67 brain metastasis tissue, n (%)**	
< 30	123 (38)
>/= 30	205 (62)
Unknown	56
**PDL1 extracranial tissue, n (%)**	

© 2022 Wasilewski et al. *JAMA Network Open.*

< 1	30 (32)
>/= 1	63 (68)
Unknown	291
**Ki67 extracranial tissue, n (%)**	
< 30	36 (40)
>/= 30	53 (60)
Unknown	295
**EGFR status of brain metastasis tissue, n (%)**	
wildtype	231 (94)
mutated	15 (6.1)
Unknown	138
**ALK status of brain metastasis tissue, n (%)**	
wildtype	213 (97)
mutated	7 (3.2)
Unknown	164
**TTF1 status of brain metastasis tissue, n (%)**	
negative	126 (33)
positive	251 (67)
Unknown	7

eTable 1C: Histopathological and biomarker-related characteristics of brain metastasis tissue at baseline. *Information on molecular characteristics (i.e. driver mutations) of primary tumor tissue was not included into tabular data - only results from brain metastasis tissue were listed.

© 2022 Wasilewski et al. *JAMA Network Open.*

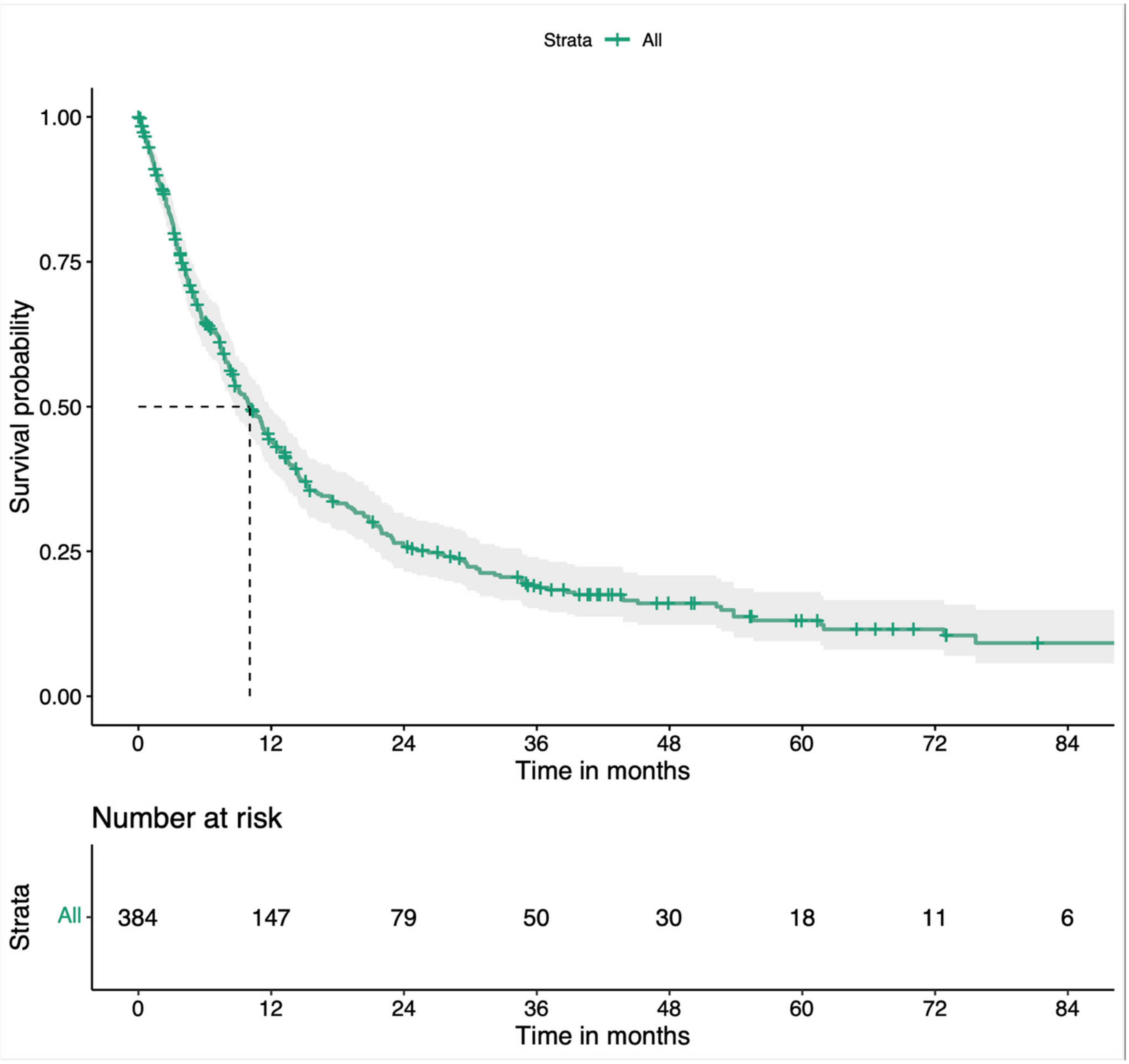

eFigure 1: Kaplan Meier survival estimates of the whole patient cohort

Kaplan Meier survival analysis for OS in the entire patient cohort of NSCLC patients subjected to craniotomy and brain metastasis removal (N=384).

© 2022 Wasilewski et al. *JAMA Network Open.*

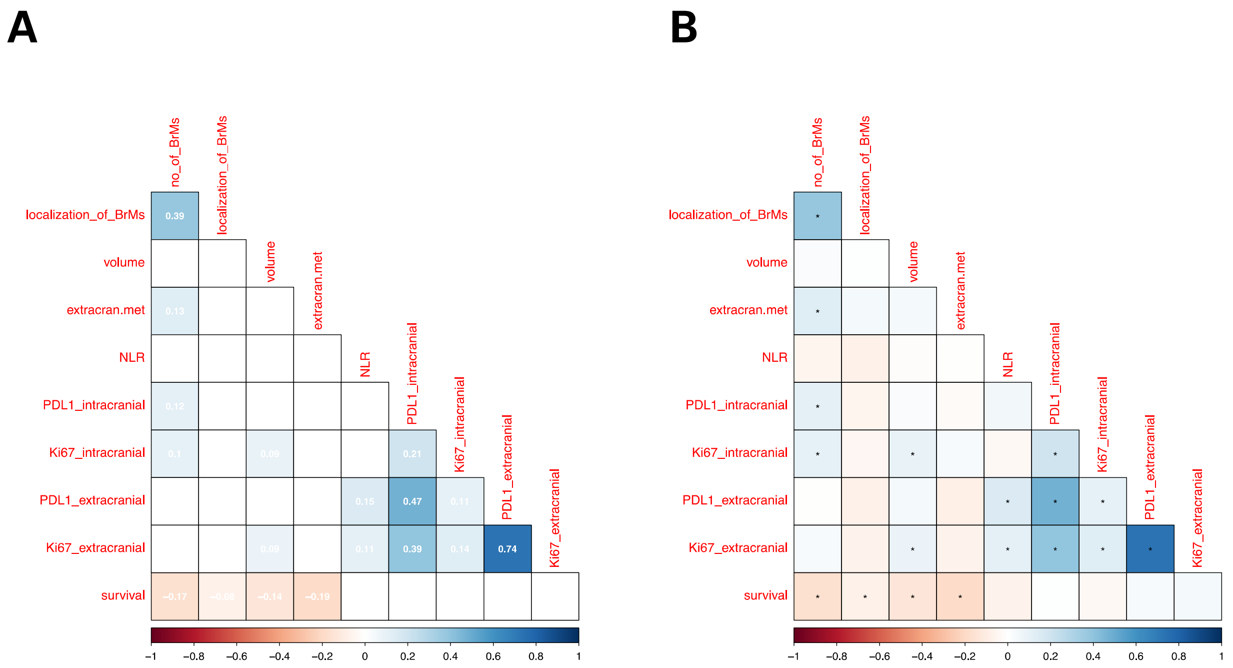

eFigure 2: Correlation matrices of radiological and biomarker-related characteristics on survival

Correlation plots using non-parametric Spearman’s correlation test to measure the degree of associatation between different clinically linked covariates, with correlation coefficients depicted in case a significant correlation was present (A). The right correlation plot shows the respective significance levels (B).

eTable 2: Univariable and multivariable Cox proportional regression in the whole patient cohort

Dependent: Surv(survival, censoring)		all	HR (CPH univar. analysis)	HR (CPH multivar.analysis)
localization_of_BrMs	supratentorial	234 (60.9)	-	-
	infratentorial	60 (15.6)	1.10 (0.80-1.51, p=0.550)	
	both	90 (23.4)	1.48 (1.12-1.95, p=0.005)	1.62 (0.95-2.77, p=0.077)
GPA2 (lung mol GPA score)	GPA below 2,5	246 (64.4)	-	-
	GPA at least 2,5	136 (35.6)	0.45 (0.35-0.57, p<0.001)	0.98 (0.60-1.58, p=0.924)

© 2022 Wasilewski et al. *JAMA Network Open.*

no_of_BrMs (number of brain metastases)	one	144 (37.5)	-	-
	two	127 (33.1)	1.39 (1.05-1.84, p=0.021)	0.89 (0.56-1.41, p=0.610)
	more than two	113 (29.4)	1.96 (1.47-2.60, p<0.001)	1.16 (0.66-2.06, p=0.604)
extracran.met (extracranial metastasis)	No	257 (66.9)	-	-
	Yes	127 (33.1)	1.81 (1.42-2.31, p<0.001)	**2.17 (1.34-3.51, p=0.002)**
primary_tumor.Rx2 (primary tumor resection)	no primary tumor resection	262 (68.2)	-	-
	primary tumor resection	122 (31.8)	0.47 (0.36-0.61, p<0.001)	**0.51 (0.32-0.82, p=0.006)**
adj.RTx3 (modality of adjuvant RTx)	other than SRS	340 (88.5)	-	-
	SRS	44 (11.5)	0.56 (0.39-0.82, p=0.002)	
adj.tx2 (adjuvant theray after neurosurgery)	RTx + CTx	108 (63.2)	-|	-|
	RTx + ICI	63 (36.8)	0.45 (0.30-0.68, p<0.001)	**0.32 (0.20-0.51, p<0.001)**

Cox regression analysis of potential prognostic covariates and overall survival for the whole patient cohort (N=384).

© 2022 Wasilewski et al. *JAMA Network Open.*

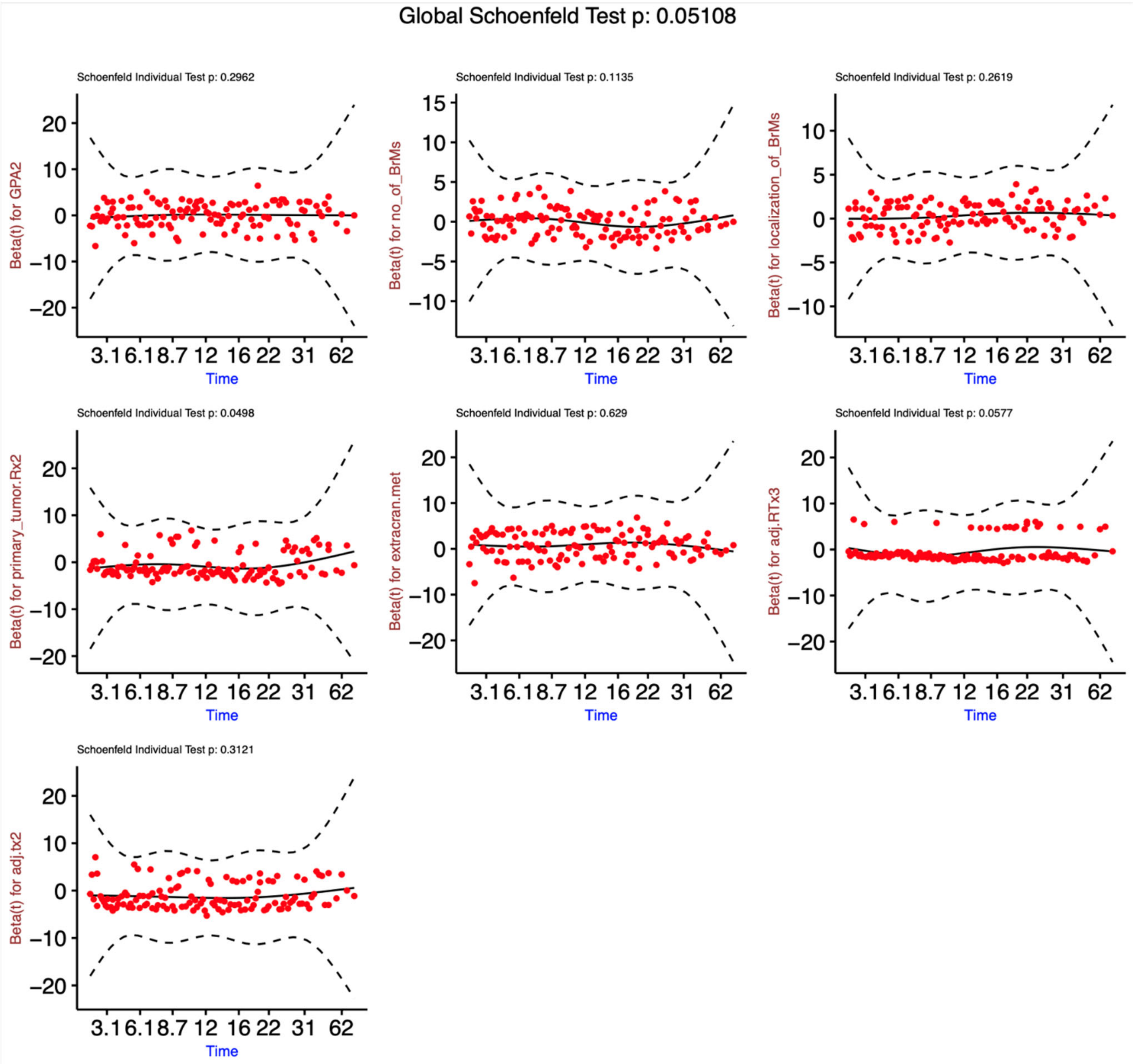

eFigure 3: Testing for the proportional hazards (PH) assumption with Schoenfeld residuals

Statistical testing including graphical diagnostics based on scaled Schoenfeld residuals for each covariate of interest (prognostic covariates): GPA score, number of brain metastases, localization of brain metastases, primary tumor resection, extracranial metastases, modality of adjuvant radiation therapy, adjuvant therapy; only adj.TTx3 (juvant radiation therapy) reached a nearly significance, hence global Schoenfeld test was not significant, therefore proportional hazards assumption was assumed.

© 2022 Wasilewski et al. *JAMA Network Open.*

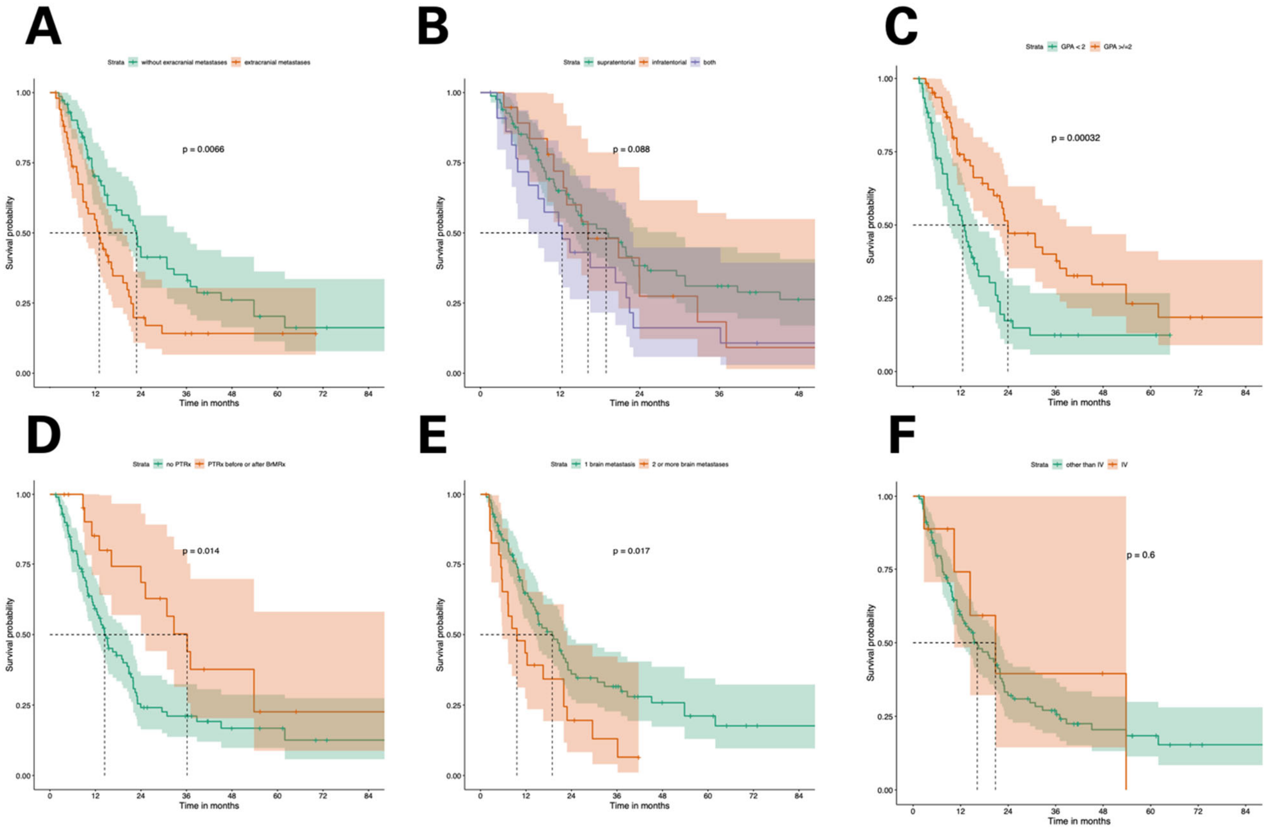

eFigure 4: Stratified Kaplan-Meier curves in matched patients for overall survival

Kaplan Meier analysis stratified according to the following characteristics: **A)** presence of extracranial metastases; **B)** localization of brain metastatic lesions at baseline (i.e. before first brain metastasis resection); **C)** graded prognostics assessment (GPA) score; **D)** status of primary tumor resection; **E)** number of brain metastasis resections; **F) UICC stage.**

© 2022 Wasilewski et al. *JAMA Network Open.*

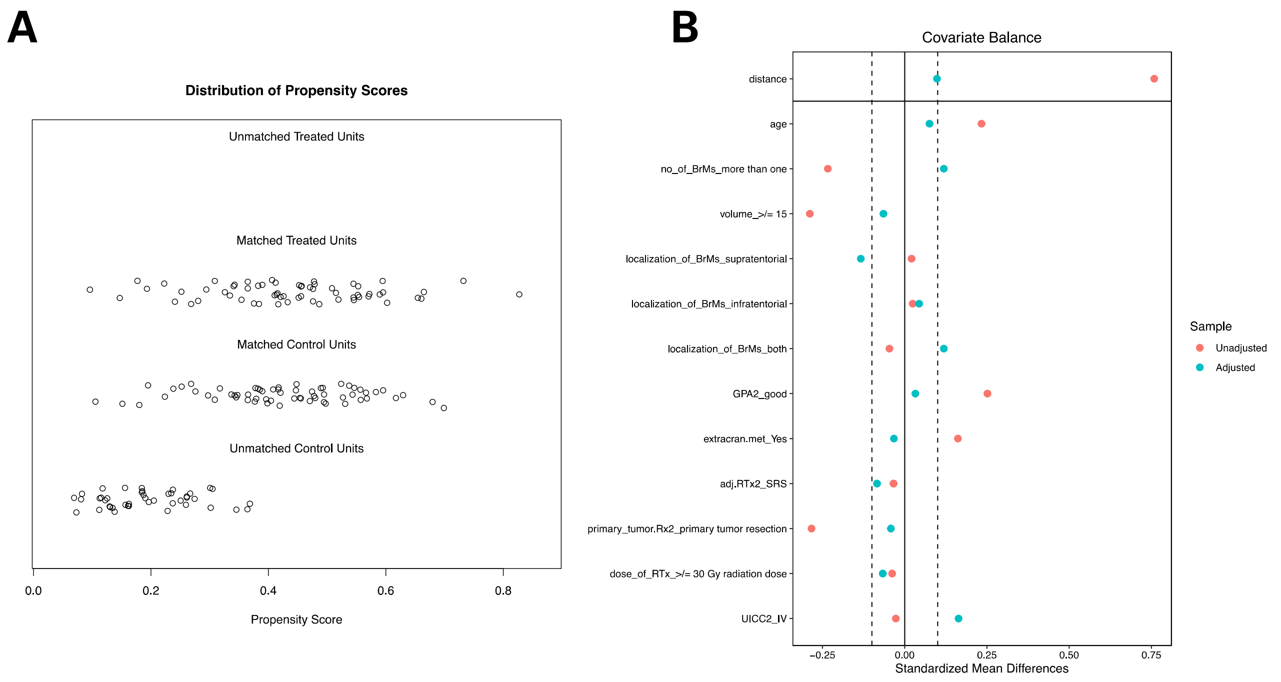

eFigure 5: Distribution of propensity scores and standardized mean differences

Distribution map after propensity score matching (PSM) (**A**). Plot displaying the standardized mean difference (SMD) of each covariate considered for PSM between treatment groups standardized (**B**). R code can be accessed upon request via Github: https://github.com/dasilew/clinicalNSCLC_1.

eTable 3: Univariable and multivariable Cox proportional regression in the matched cohort

explanatory		HR (CPH univar. analysis)	HR (CPH multivar.analysis)
gender	Male<br>Female	1.00 (0.65-1.54, p=0.996)	
age		1.02 (1.00-1.05, p=0.077)	
GPA2 (GPA score)	Bad GPA (2 or less)<br>Good GPA (>/= 2)	0.48 (0.31-0.74, p=0.001)	**0.46 (0.22-0.99, p=0.047)**
extracranial metastases	No<br>Yes	1.88 (1.22-2.91, p=0.004)	1.02 (0.49-2.12, p=0.961)
volume	< 15<br>>/= 15	0.98 (0.64-1.51, p=0.932)	
Number of_brain metastases	One<br>>/= 1	1.84 (1.11-3.06, p=0.019)	1.30 (0.64-2.65, p=0.471)

© 2022 Wasilewski et al. *JAMA Network Open.*

Localization of brain metastasis	infratentorial	1.35 (0.75-2.44, p=0.322)	
Localization of brain metastasis	Both (supra-/infratentorial)	1.71 (1.00-2.93, p=0.052)	
UICC stage	Stage IV	0.79 (0.32-1.95, p=0.610)	
Primary tumor resection	No<br>Yes	0.40 (0.20-0.78, p=0.007)	**0.26 (0.12-0.53, p<0.001)**
Adjuvant RTx (SRS vs. other modalities)	SRS	0.64 (0.36-1.11, p=0.113)	
Adjuvant RTx + ICIs vs. RTx + CTx		0.44 (0.28-0.69, p<0.001)	**0.27 (0.16-0.45, p<0.001)**

Univariable and multivariable Cox proportional regression analysis for matched data (N=124).

© 2022 Wasilewski et al. *JAMA Network Open.*
